# Improving Lone Work Practice Within a Mental Health Trust: A Quality Improvement Project

**DOI:** 10.1192/bjo.2025.10384

**Published:** 2025-06-20

**Authors:** Tooba Khan, Khunsha Cheema, Yuyu Htwe, Hannah Liu, Anum Asim

**Affiliations:** 1 Birmingham and Solihull Mental Health Foundation Trust, Birmingham, United Kingdom; 2 Birmingham Women’s and Children’s Trust, Birmingham, United Kingdom

## Abstract

**Aims:** Ensuring resident doctors’ safety during lone working is crucial, due to unique risks and challenges faced when working alone. Birmingham and Solihull Mental Health Foundation Trust’s (BSMHFT) current lone working policy recommends local procedures based on risk assessment and site needs. However, gaps in implementation have raised concerns about the consistency and effectiveness of safety measures.

Aims were to:

1. Increase awareness and adherence to lone working policy amongst Resident doctors in inpatient and community settings by 20% by September 2024.

2. Standardise lone working processes across BSMHFT by September 2024.

**Methods:** Our quality improvement (QI) project worked alongside the Trust’s QI team, utilising improvement methodology. A baseline survey was conducted to understand issues faced whilst lone working, alongside process mapping to analyse root cause. We followed the Model For Improvement model and initiated four Plan-Do-Study-Act (PDSA) cycles for the following interventions:

Incorporated Lone Work checklist into orientation checklist for all resident doctors rotating within Trust.

Lone work presentation at induction.

Created video on lone working, alarm use and policy guidance.

Sent clinical supervisors reminders to discuss lone work procedures with their trainees.

Data was collected via surveys alongside video views and returned checklists.

**Results:** 4 surveys were conducted amongst resident doctors in BSMHFT.

Before interventions:

Baseline survey (24 responses): 71% conducted lone working. 29% felt informed about policies, 43% received alarms with 66% of these trained to use them.

First pulse check survey (25 responses): 8% felt very confident in lone working, 32% had alarms, and 32% were “not confident” in following trust policies.

After interventions:

Second pulse check survey (17 responses): confidence improved with 35% feeling very confident, 65% had alarms, and all could follow trust policies.

Detailed post-intervention survey (19 responses): 68% conducted lone working, 63% felt well informed and received alarms, 72% felt confident using alarms.

Feedback on interventions:

83% found the lone working video guide helpful.

68% were unaware of or had incomplete induction checklists for local lone working policies.

**Conclusion:** We have been able to achieve our aim of improving adherence and awareness of lone working policy amongst resident doctors by over 20% (33.83%). Alongside, there is improvement in doctors’ confidence in lone working and the number, and utilisation, of alarms issued. This cycle has highlighted ongoing challenges and a need for further PDSAs to continue to improve, for example, pathway of escalation for lone working incidents and named alarms for doctors. The second cycle commences March 2025.

